# Two new species of the genus *Microplitis* Förster, 1862 (Hymenoptera, Braconidae, Microgastrinae) from China

**DOI:** 10.3897/zookeys.859.31720

**Published:** 2019-07-02

**Authors:** Wangzhen Zhang, Dongbao Song, Jiahua Chen

**Affiliations:** 1 Plant Protection College, Fujian Agriculture and Forestry University, Fuzhou 350002, China; 2 Fuzhou Airport Inspection and Quarantine Bureau, Changle 350209, China; 3 Institute of Beneficial Insects, Fujian Agriculture and Forestry University, Fuzhou 350002, China

**Keywords:** Braconidae, taxonomic key, *
Microplitis paizhensis
*, *
Microplitis bomiensis
*

## Abstract

Two new species of *Microplitis* Förster, 1862, *M.bomiensis* Zhang, **sp. nov.**, and *M.paizhensis* Zhang, **sp. nov.** from Tibet, China are described and illustrated. A key to the species of the genus *Microplitis* Förster from China is added.

## Introduction

The genus *Microplitis* Förster was established by Förster (1862) with the type species *Microgastersordipes* (Nees von Esenbeck, 1834).

In 1982, van Achterberg examined three male specimens of *Ichneumondeprimator*, and found that the genus *Microplitis* should not be *Microgaster*, but rather *Microplitis* (van Achterberg 1982). Mason (1981) and Whitfield (1987) suggested and recommended to the International Committee of Zoological Nomenclature (ICZN) to abandon *Ichneumondeprimator* as the type species of *Microgaster*, and reassigned *Microgasteraustralis* Thomson, 1895 as type species of this genus; the original genus names of *Microplitis* and *Microgaster* remained unchanged. This recommendation was adopted by ICZN in 1988 (International Commission on Zoological Nomenclature 1988).

*Microplitis* is a moderately large genus in Microgastrinae, with 190 species known from all over the world, of which 37 species have been reported from China (Fernandez-Triana and Ward 2015; Zhang et al. 2017).

This paper describes and illustrates two new species.

## Materials and methods

This study is based on a collection of specimens preserved in the Parasitic Hymenoptera Collection of the Institute of Beneficial Insect, College of Plant Protection, Fujian Agriculture and Forestry University (FAFU; Fuzhou, China). The morphological characters were examined and photographed using a Leica M205C digital stereomicroscope. All specimens described are deposited in the Beneficial Insects Institute, Fujian Agriculture and Forestry University (Fuzhou, China). The morphological terminology used in this paper follows van Achterberg (1988) and Austin and Dangerfield (1992, 1993). Terminology for wing venation is based on the modified Comstock-Needham system (Eady 1974; van Achterberg 1979). Abbreviations used in this paper are as follows: POL, Postocellar line (minimum distance between posterior ocelli); OD, Posterior ocellus maximum diameter; OOL, Ocular-ocellar distance (minimum distance between posterior ocellus and eye); T1, T2, etc., first, second, etc. metasomal tergites.

## Taxonomic part

### Microplitis

Taxon classificationAnimaliaHymenopteraBraconidae

Förster, 1862

Microplitis Förster, 1862: 245 [type species, by original designation, Microgastersordipes Nees ab Esenbeck, 1834.] Nixon 1970: 3. Mason 1981: 132. Austin and Dangerfield 1992 [see Shenefelt (1973: 737) for complete bibliography].
Dapsilotoma Cameron, 1906: 101 [type species, by monotypy, Dapsilotomatestaceipes Cameron, 1906]. Synonymized by Viereck (1914: 25).
Glabromicroplitis Papp, 1979: 176 [type species, Glabromicroplitismahunkai Papp, 1979]. Synonymized by Austin and Dangerfield (1992).

#### Diagnosis.

Hypopygium usually small, never bearing longitudinal creases along median line. Ovipositor and sheaths usually projecting only a little beyond apex of hypopygium; sheaths bearing a few setae distally. T1 variable from wide to narrow apically and usually moderately sculptured; T2 rarely weakly sculptured and often with a weakly delimited trapezoidal median area; T3 longer than T2, the transverse groove between them poor; remaining tergites nearly smooth. Propodeum usually convexly rounded and often with a distinct percurrent medial keel, never with an areola, surface almost completely rugose, sometimes reticulo-rugose. Mesoscutum often densely sculptured, sometimes smooth, and with notauli, sometimes strongly defined. Posterior band of scutellum usually smooth but interrupted medially by rugosity. Fore wing usually with a D-shaped areolet, shape variable in some species, subtriangular, rectangular, etc.; 1CU1 much shorter than 2CU1; r short. Hind wing with vannal lobe convex and fringed throughout. Hind coxa small and not longer than T1; hind spurs shorter than half length of basitarsus. Labial palpi 3-jointed, sometimes 4-jointed.

Generally, the genus are clearly distinct from other genera. A detailed description of the genus and references to the revised generic diagnosis and Oriental *Microplitis* species can be made using the most recent data (Mason 1981; Austin and Dangerfield 1993; Ranjith et al. 2015).

### Key to species of the genus *Microplitis* Förster from China

**Table:** 

1	T1 less than 1.5× as long as maximum width	**2**
–	T1 more than 1.5× as long as maximum width	**4**
2	Hypopygium in ventral view apically emarginated	***M.ocellatae* (Bounche)**
–	Hypopygium in ventral view not emarginated apically	**3**
3	Head 2.1× as wide as long in dorsal view; antennae as long as body	***M.amplitergius* Xu & He**
–	Head less than 1.9× as wide as long in dorsal view; antennae distinctly longer than body	***M.hirtifacialis* Song & You**
4	Notauli virtually absent, indicated only by indentations, or shallow; mesoscutum weakly punctate or simply sculptured	**5**
–	Notauli impressed, percurrent and meeting posterioly, or deep; mesoscutum roughly punctate or with rugose sculpture	**10**
5	Propodeum with basal transverse carina distinct	***M.carinata* Ashmead, 1900**
–	Propodeum with basal transverse carina indistinct or absent	**6**
6	Head in dorsal view broadening behind eye; T1 less than 1.8× as long as maximum width; tegula black	**7**
–	Head in dorsal view not broadening behind eye; T1 more than 2× as long as maximum width; tegula reddish yellow	**9**
7	Areolet approximately triangular; stigma with basal patch semihyaline	***M.basipallescentis* Song & Chen**
–	Areolet approximately quadrangular or rectangular; stigma without basal patch Semihyaline	**8**
8	Mesosoma narrower than head; T1 slightly narrowed in posterior part; 1-R1 1.7× as long as the distance from itself to apex of marginal cell	***M.fujianica* Zhang, Song et Chen**
–	Mesosoma wider than head; T1 slightly widened in posterior part; 1-R1 2.1× as long as the distance from itself to apex of marginal cell	***M.longwangshanus* Xu & He**
9	Vein 1-R1 (metacarpus) 1.6× as long as its distance from apex of marginal cell and 1.3× as long as stigma	***M.bomiensis* , sp.n.**
–	Vein 1-R1 (metacarpus) 1.1× as long as its distance from apex of marginal cell and 0.7× as long as stigma	***M.helicoverpae* Xu & He**
10	T1 distinctly broadening posteriorly	**11**
–	T1 either weakly broadening posteriorly, or subparallel to parallel sides	**17**
11	Scutellum evenly or almost evenly rugose	**12**
–	Scutellum anteriorly or antero-medially smooth with weak and rather scattered punctures	**13**
12	Flagellomeres thick and dark brown; 1-R1 1.5× as long as the distance from itself to apex of marginal cell	***M.crassiantenna* Song & Chen**
–	Flagellomeres thin and reddish yellow; 1-R1 2× as long as the distance from itself to apex of marginal cell	***M.tadzhica* Telenga**
13	T2 rugose or at least shrivelled medially	***M.menciana* Xu & He**
–	T2 smooth or at most slightly uneven	**14**
14	Both outer and inner spurs are the same length, only 0.2× as long as basitarsi; propodeum with basal transverse carina distinct	***M.brevispina* Song & Chen**
–	Both outer and inner spurs are equal or unequal length, more than 0.2× as long as basitarsi; propodeum with basal transverse carina indistinct	**15**
15	Stigma fully dark or reddish brown, without pale basal spot	***M.borealis* Xu & He**
–	Stigma blackish with a yellow basal spot at its proximal third	**16**
16	1-R1 almost equal to stigma; tegula reddish yellow	***M.jiangsuensis* Xu & He**
–	1-R1 half as long as stigma; tegula black	***M.cubitellanus* Xu & He**
17	T1 more than 1.7× as long as maximum width; usually with subparallel or parallel sides	**18**
–	T1 less than 1.7× as long as maximum width; usually more or less broadening posteriorly, or subquadrate	**26**
18	Flagellum reddish yellow to yellow white basely, dull apically, or blackish basely, reddish yellow apically	**19**
–	Flagellum back or brownish yellow entirely	**20**
19	Antenna short, clearly shorter than body	***M.chui* Xu & He**
–	Antenna long, clearly as long as or longer than body	***M.zhaoi* Xu & He**
20	Head in dorsal view 2 or more than 2× as broad as long	**21**
–	Head in dorsal view less than 1.8× as broad as long	**24**
21	Middle and hind femora mostly or entirely black or blackish brown	**22**
–	Middle and hind femora mostly or entirely reddish yellow	**23**
22	Mesonotum antero-medially dull with dense sculpture; fore wing slightly hyaline	***M.bicoloratus* Xu & He**
–	Mesonotum antero-medially shiny with few fine punctures; fore wing almost opaque	***M.obscuripennatus* Xu & He**
23	Hind coxa black	***M.marshalli* Kokujev**
–	Hind coxa reddish yellow	***M.longiradiusis* Xu & He**
24	Metasoma usually reddish yellow or testaceous, or T1 and last 2 or 3 segments blackish; hind coxa reddish yellow	***M.pallidipes* Szépligeti**
–	Metasoma black, or T2–3 reddish yellow to brownish yellow; hind coxa black	**25**
25	T2–3 reddish yellow to brownish yellow	***M.mediator* Haliday**
–	T2–3 brownish testaceous to black	***M.tuberculifer* Wesmael**
26	Hind femora mostly or entirely black	**27**
–	Hind femora mostly or entirely reddish yellow to brownish yellow	**30**
27	Wings with pale brown areas over first discal cell and above areolet	***M.prodeniae* Rao & Kurian**
–	Wings without pale brown areas over first discal cell and above areolet, or only with brown area above areolet	**28**
28	Tegula reddish yellow; stigma blackish brown; hind tibia with basal white or yellowish white ring	**29**
–	Tegula black; stigma blackish brown with yellow basal spot at its proximal third; hind tibia reddish yellow	***M.varipes* Ruthe**
29	Antennae distinctly longer than body; hind tibia yellow	***M.paizhensis* sp. nov.**
–	Antennae slightly longer than body; hind tibia yellowish white	***M.albotibialis* Telenga**
30	Fore wing with areolet approximately triangular	***M.strenuus* Reihard**
–	Fore wing with areolet approximately quadrangular	**31**
31	T1 slightly widened towards apex; antennae with flagellomeres 12–15 tightly connected	***M.changbaishanus* Song & Chen**
–	T1 parallel or subparallel-sided; antennae with flagellomeres 12–15 loosely connected	**32**
32	Penultimate joint of antenna 2.5 times as long as wide, apex of hypopygium ending far beyond apex of abdomen	***M.leucaniae* Xu & He**
–	Penultimate joint of antenna 1.6–2.0 times as long as wide, apex of hypopygium reach beyond apex of abdomen	***M.vitellipedis* Li, Tan et Song**

### Microplitis paizhensis

Taxon classificationAnimaliaHymenopteraBraconidae

Zhang sp. nov.

http://zoobank.org/94F03DB7-B4AC-4293-B7D2-D7992DC54AC9

Figs 1–7

#### Etymology.

The specific name is derived from the type locality.

#### Type material.

Holotype: female, Paizhen, Tibet, 94°58'10.57"Е, 29°50'45.67"Х, 3696 m, 16.vii.2013, leg. Zhang Wangzhen (FAFU).

#### Comparative diagnosis.

This species is similar to *Microplitisfujianica* Song and Zhang, but can be distinguished by its shiny pronotum, which is sparsely punctate (vs rugose-punctate); fore wing with vein 1R-1 (metacarpus) 1.3× as long as its distance from apex of marginal cell (vs vein 1-R1 1.7× as long as its distance from apex of marginal cell); T2 subrectangular, ratio of apical width: central length = 3.2: 0.7 (vs T2 nearly triangular, ratio of apical width: central length = 3.6: 1.4).

This species (*M.paizhensis*, sp. nov.) is similar to *M.albotibialis* Telenga, but can be distinguished by antennae distinctly longer than body (vs antennae slightly longer than body); hind tibia yellow (vs hind tibia yellowish white). Frons faintly sculptured (vs frons coarsely sculptured). POL: OD = 1.0: 0.4 (vs POL: OD: OOL = 2.0: 2.0).

This species is also similar to *Microplitisbomiensis*, sp. nov. (see below for further diagnosis).

#### Description.

Female (holotype).

Head. Roughly triangular in anterior view, with antennal sockets high above the middle level of the eyes. Face slightly convex, finely micropunctate associated with long setae. Inner margin of the eyes straight to moderately emarginate near antennal sockets. Transverse in dorsal view, 1.7× as wide as long, posterior vertex and temples finely punctate to rugose-punctate, with long sparse setae. Frons faintly sculptured. Ocelli small, in a high triangle, imaginary tangent of posterior margin of anterior ocellus far from posterior ocelli. POL: OD: OOL = 1.0: 0.4: 0.9. Antennae longer than body (14.2: 10.5), flagellomeres thin, setose. Flagellomere proportion: 2 L/W (section 2 length/ width) = 2.3, 8 L/W = 2.4, 14 L/W = 2.6. L 2/14 = 1.2, W 2/14 = 1.4. F12–15 (Flagellomere 12–15) loosely connected.

Mesosoma. Mesosoma almost as wide as head. Pronotum shiny, sparsely punctate. Mesoscutum evenly and densely punctate, setose. Notauli shallow. Scutellar lunules deep, broad, divided by five carinae. Disc of scutellum shiny, weakly convex, evenly punctate, with white setae, its rugose-punctate spot in the middle interrupting the posterior, polished band of scutellum. Propodeum rather evenly curved, coarsely reticulate-rugose, with a median longitudinal carina.

Wings. Fore wing: vein 1-R1 (metacarpus) 1.3 × as long as its distance from apex of marginal cell and 1.1 × as long as stigma. Vein r (1^st^ radius) arising distally from the middle of the stigma and approximately as long as 2-SR. Areolet approximately quadrangular. Stigma 2.9× as long as width. Width of 1^st^ discal cell: height of 1^st^ discal = 20.0: 21.5. 1-CU1: 2-CU1: m-cu = 7.5: 11.0: 10.0. Hind wing vein cu-a slightly incurved.

Legs. Hind coxa small, slightly shorter than T1. Inner hind tibial spur almost as long as outer one, about 0.3× as long as hind basitarsus.

Metasoma. Slightly longer than mesosoma (5.3: 4.8). T1 widening towards apex, then narrowing to the extreme apex, weakly punctured except for moderately depressed base and small apical swelling smooth. T2 subrectangular, smooth, ratio of apical width: central length = 3.2: 0.7, its median field slightly raised. T3 longer than T2 (1.0: 0.7), suture between T3 and T2 weak, T3 and the remaining tergites smooth, shiny, sparsely setose. Hypopygium small, slightly shorter than tip of metasoma; ovipositor sheath short, approximately 1.3× as long as second hind tarsomere.

Color. Black. Antennae dark brown. Maxillary palps, labial palps, and tibial spur pale yellow. Ocelli reddish. Stigma and most veins brown, semitransparent. Wings hyaline without infuscations, except for light brown central area. Wing setae whitish. Legs yellow except all coxae, basal 2/5 of fore femur, basal 4/5 of mid femur, hind femur black, distal 2/5 of hind tibia and tarsus brown. Metasoma blackish brown except for T1 and T2 which are black.

Body length 3.2 mm; fore wing length 3.8 mm.

Male. Unknown.

#### Distribution.

Tibet, China.

#### Habitat.

Prairie and bushes.

**Figure 1–7.**
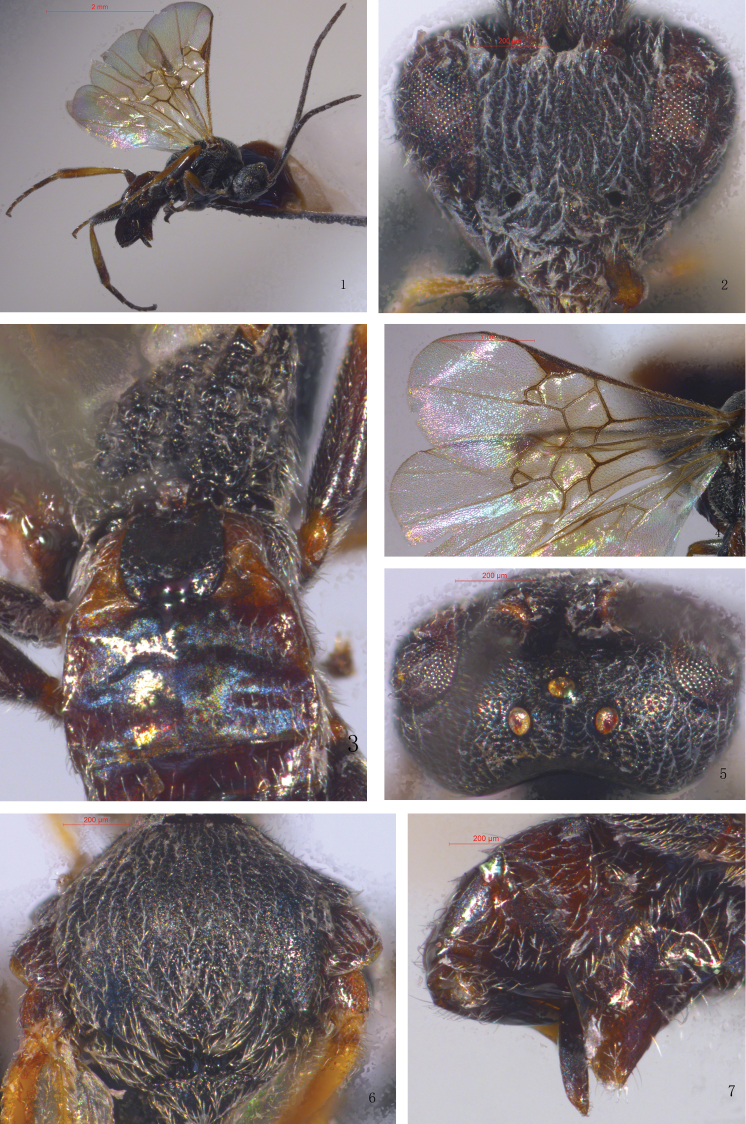
*Microplitispaizhensis*, sp. nov. (female) **1** Habitus, lateral view **2** Head, anterior view **3** Propodeum and basal tergites of metasoma **4** Wings **5** Head, dorsal view **6** Mesoscutum **7** Apex of metasoma (showing ovipositor).

### Microplitis bomiensis

Taxon classificationAnimaliaHymenopteraBraconidae

Zhang sp. nov.

http://zoobank.org/55F4D31C-13EC-4856-B38A-4B58B7FD92EB

Figs 8–14

#### Etymology.

The specific name “*bomiensis*” is derived from the type locality.

#### Type material.

Holotype: female, Bomi, Tibet, 96°23'23.23"E, 29°36'22.33"N, 3427 m, 28.vii. 2013. Leg. Zhang Wangzhen (FAFU).

#### Comparative diagnosis.

Morphologically this species and *M.paizhensis* Zhang, sp. nov. are very similar; the main points of distinction are to be found in the former having golden setae on mesoscutum and disc of scutellum (vs light grey or colourless setae on mesoscutum and disc of scutellum). Fore vein 1-R1 1.6× as long as its distance from apex of marginal cell and 1.3× as long as stigma (vs. vein 1-R1 1.3× as long as its distance from apex of marginal cell and 1.1× as long as stigma). Mid coxa reddish brown, hind coxa black brown or infuscate (vs all coxae black).

The new species is also similar to *M.helicoverpae* Xu & He with the distinction between them as following: vein 1-R1 1.6× as long as its distance from apex of marginal cell and 1.3× as long as stigma (vs vein 1-R1 1.1× as long as its distance from apex of marginal cell and 0.7× as long as stigma). Areolet approximately quadrangular (vs areolet approximately triangular). T1 2.2× as long as wide (vs T1 1.7× as long as wide).

#### Description.

Female (holotype).

Head. Subcircular in anterior view, lateral temples hidden behind eyes in anterior view. Width of face (at widest) half as wide as head. Face flat to slightly convex, densely punctate, with associated dense setae. Inner margin of eyes straight to moderately emarginate adjacent to antennal sockets. Eyes setose. Transverse in dorsal view, 2.2× as wide as long. Ocelli medium-sized, in a high triangle, imaginary tangent of posterior margin of anterior ocellus distant from posterior ocelli. Vertex shiny, shallowly punctate. Frons depressed, nearly smooth. POL: OD: OOL = 0.9: 0.4: 1.1. Antennae long than body (14.1: 10.3), flagellomeres thin, with bristly setae. Flagellomere proportion: 2 L/W (Flagellomere 2 length/ width) = 2.5, 8 L/W = 2.6, 14 L/W = 2.5. L 2/14 = 1.4, W 2/14 = 1.3. F12–15 (Flagellomere 12–15) loosely connected.

Mesosoma. Thorax slightly wider than head (7.3: 7.8). Pronotum sparsely punctae. Mesoscutum shiny, evenly punctate, with dense setae. Notauli faintly impressed. Scutellar lunules broad, divided by five carinae. Disc of scutellum shiny, weakly convex, evenly punctate, with setae, its rugose spot in the middle interrupting the posterior, polished band of scutellum. Propodeum rather evenly curved in profile, coarsely reticulate and rugose, with a median longitudinal carina.

Wings. Fore wing: vein 1-R1 (metacarpus) 1.6× as long as its distance from apex of marginal cell and 1.3 × as long as stigma. Vein r (1^st^ radius) emitted distally from middle of stigma and approximately as long as 2-SR. Areolet approximately quadrangular. Stigma 2.9× as long as wide. Ratio of width of 1^st^ discal cell: height of 1^st^ discal = 21.6: 17.5. 1-CU1: 2-CU1: m-cu = 7.4: 11.5: 9.5. Hind wing: vein cu-a incurved.

Legs. Hind coxa small, slightly shorter than T1. Inner hind tibial spur almost as long as outer one, 0.3× as long as hind basitarsus. Metasoma Slightly shorter than mesosoma (4.9: 5.2). T1 2.2× as long as wide, parallel-sided, with broad shallow medial depression on basal 1/3, surface rugulose except for smooth apical swelling. T2 subtrapezoidal, its apical width: medial length ratio = 3.1: 0.9, smooth, shiny, glabrous, with a shield-shaped median field indicated by oblique grooves. T3 longer than T2 (1.3: 0.9), suture between T3 and T2 reduced to slight depression. T3 and the following tergites smooth, each with one or two transverse rows of sparse hairs posteriorly, denser laterally. Hypopygium small. Ovipositor sheath short, 1.3× as long as second hind tarsomere.

Color. Body generally black to dark brown. Palps yellow to white. Setae of mesoscutum and disc of scutellum golden. Lateral edges of T1–T3 reddish yellow. Hypopygium reddish brown. Antennae dark brown or brown. Wings hyaline, venation brown, stigma with pale yellowish patch basally. Legs yellow, except mid coxa which are reddish brown; hind coxa, tibia, and tarsus black brown or infuscate.

Body length 3.4 mm; forewing length 3.9 mm.

Male. Unknown.

#### Distribution.

Tibet, China.

#### Habitat.

Prairie and bushes.

**Figure 8–14.**
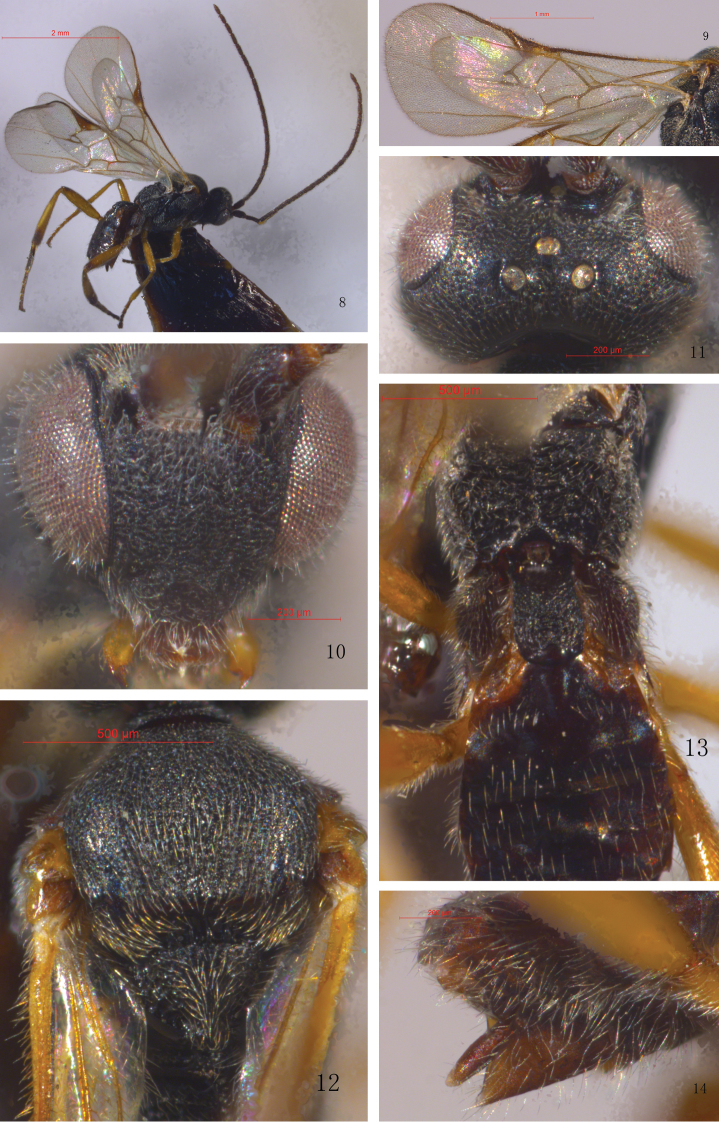
*Microplitisbomiensis*, sp. nov. (female) **8** Habitus, lateral view **9** Wings **10** Head, anterior view **11** Head, dorsal view **12** Mesoscutum and scutellum **13** Propodeum and basal tergites of metasoma **14** Apex of metasoma (showing ovipositor).

## Remarks

Both new species were collected in high-altitude areas in Tibet, China (above 3400 m), which is relatively rare for this group above this altitude. We also collected single male specimen of a third species, which, considering the importance of the females in microgastrine taxonomy and the recommendation of the reviewers, will not be published for the time being.

## Supplementary Material

XML Treatment for Microplitis

XML Treatment for Microplitis paizhensis

XML Treatment for Microplitis bomiensis

## References

[B1] Austin AD Dangerfield PC (1989) The taxonomy of New World microgastrine braconids (Hymenoptera) parasitic on *Diatraea* spp. (Lepidoptera: Pyralidae). Bulletin of Entomological Research 79(1): 131–144. 10.1017/s0007485300018642

[B2] Austin AD Dangerfield PC (1992) Synopsis of Australasian Microgastrinae (Hymenoptera : Braconidae), with a key to genera and description of new taxa. Invertebrate Taxonomy 6(1): 1–76. 10.1071/it9920001

[B3] Austin AD Dangerfield PC (1993) Systematics of Australian and New Guinean *Microplitis* Foerster and Snellenius Westwood (Hymenoptera: Braconidae : Microgastrinae), with a review of their biology and host relationships. Invertebrate Taxonomy 7(5): 1097–1166. 10.1071/it9931097

[B4] Cameron P (1906) On the Tenthredinidae and parasitic Hymenoptera collected in Baluchistan by Major C.G. Nurse. Part I. Journal of the Bombay Natural History Society 17: 89–107. https://biodiversitylibrary.org/page/30119169

[B5] Chen JH Ji QE Song DB (2004) A new species of *Microplitis* Förster from China (Hymenoptera: Braconidae: Microgastrinae). Entomological Journal of East China 13(2): 1–5. http://med.wanfangdata.com.cn/Paper/Detail/PeriodicalPaper_hdkcxb200402001

[B6] Eady RD (1974) The present state of nomenclature of wing venation in the Braconidae (Hymenoptera); its origins and comparison with related groups. Journal of Entomology Series B, Taxonomy 43(1): 63–72. 10.1111/j.1365-3113.1974.tb00089.x

[B7] Fernandez-Triana J Ward D (2015) Microgastrinae Wasps of the World. http://microgastrinae.myspecies.info/ [Accessed on: 2019-5-23]

[B8] International Commission on Zoological Nomenclature (1988) Opinion 1510. *Microgaster* Latreille, 1804 (Insecta, Hymenoptera): *Microgasteraustralis* Thomson, 1895 designed as the type species. Bulletin of Zoological Nomenclature 45: 239–240. https://biodiversitylibrary.org/page/12229669

[B9] Mason WRM (1981) The polyphyletic nature of *Apanteles* Foerster (Hymenoptera: Braconidae): a phylogeny and reclassification of Microgastrinae Memoirs of the Entomological Society of Canada 113(S115): 1–147. 10.4039/entm113115fv

[B10] Nixon GEJ (1970) A revision of the N.W. European species of Microplitis Förster (Hymenoptera: Braconidae). Bulletin of the British Museum (Natural History) 25(1): 1–30. https://www.biodiversitylibrary.org/item/19402#page/6/

[B11] Ranjith AP Rajesh KM Nasser M (2015) Taxonomic studies on Oriental *Microplitis* Foerster (Hymenoptera: Braconidae, Microgastrinae) with description of two new species from South India. Zootaxa 3963(3): 369–415. 10.11646/zootaxa.3963.3.4 26249405

[B12] Song D Chen J (2004) A study on *Microgaster* Latreille from China with description of a new species (Hymenoptera: Braconidae: Microgastrmae). In: Rajmohana K Sudheer K Girish Kumar P Santhosh S (Eds) Perspectives on Biosystematics and Biodiversity: Prof T C Narendran Commemortive Volume.Systematic Entomology Research Scholars Association, Kerala, 315–325.

[B13] Song D Chen J (2008) Five new species of the genus *Microplitis* (Hymenoptera: Braconidae: Microgastrinae) from China. Florida Entomologist 91(2): 283–293. 10.1653/0015-4040(2008)91[283:fnsotg]2.0.co;2

[B14] Tobias VI (1986) Hymenoptera Fauna USSR. Guide to the Insect of European Part of the USSR 4: 344–459

[B15] van Achterberg C (1979) A revision of the subfamily Zelinae auct. (Hymenoptera, Braconidae). Tijdschrift voor Entomologie 122: 241–479. https://biodiversitylibrary.org/page/28227686

[B16] van Achterberg C (1982) Notes on some type species described by Fabricius of the subfamilies Braconidae, Rogadinae, Microgastrinae and Agathidinae (Hymenoptera: Ichneumonidae). Entomologische Berichten 42: 133–139. https://biodiversitylibrary.org/page/57854343

[B17] van Achterberg C (1988) Revision of the subfamily Blacinae Förster (Hymenoptera, Braconidae). Zoologische Verhandelingen 249: 1–324. https://pdfs.semanticscholar.org/d04d/7f072d970e54bd36e28f1c0be8b43b4a98c9.pdf

[B18] van Achterberg C (1997) Notes on the types and type depositories of Braconidae (Insecta: Hymenoptera) described by T.C. Narendran and students. Zoologische Mededelingen 71(16): 177–179. https://www.repository.naturalis.nl/document/149965

[B19] Whitfield JB (1987) Comment on the proposed designation of Microgasteraustralis Thomson, 1895 as type of Microgaster Latreille, 1804 (Insecta, Hymenoptera) (Case 2397). Bulletin of Zoological Nomenclature 47(1): 47 https://biodiversitylibrary.org/page/12229352

[B20] Whitfield JB (1995) Annotated checklist of the Microgastrinae of North America north of Mexico (Hymenoptera: Braconidae). Journal of the Kansas Entomological Society 68(3): 245–262. https://www.jstor.org/stable/25085593

[B21] Wilkinson DS (1930) A revision of the Indo-Australian species of the genus *Microplitis* (Hym. Bracon.). Bulletin of Entomological Research 21(1): 23–27. 10.1017/s0007485300021519

[B22] Xu WA He JH (1999a) A new species of Microplitis Förster (Hymenoptera: Braconidae: Microgastrinae) from Fujian, China. Entomotaxonomia 21(1): 64–68. http://www.cqvip.com/qk/96329X/199901/3424292.html

[B23] Xu WA He JH (1999b) A new species of *Microplitis* Förster from China (Hymenoptera: Braconidae, Microgastrinae). Entomological Journal of East China 8(1): 1–3. http://www.cnki.com.cn/Article/CJFDTotal-HDKC199901000.htm

[B24] Xu WA He JH (2000a) A new species and a new record of *Microplitis* Foerster from China (Hymenoptera:Braconidae:Microgastrinae). Acta Entomologica Sinica 43(2): 193–197. 10.3321/j.issn:0454-6296.2000.02.013

[B25] Xu WA He JH (2000b) A new species and a new record species of *Microplitis* Förster (Hymenoptera: Braconidae: Microgastrinae) from China. Entomological Journal of East China 9(2): 5–8. http://www.cnki.com.cn/Article/CJFDTotal-HDKC200002001.htm

[B26] Xu WA He JH (2000c) A new species of *Microplitis* Förster from China (Hymenoptera, Braconidae, Microgastrinae). Acta Zootaxonomica Sinica 25(2): 195–198. 10.3969/j.issn.1000-0739.2000.02.016

[B27] Xu WA He JH (2000d) Two new species of *Microplitis* Foerster (Hy-menoptera: Braconidae) from China. Insect Science 7(2): 107–112. 10.1111/j.1744-7917.2000.tb00346.x

[B28] Xu WA He JH (2000e) Two new species of *Microplitis* Förster (Hymenoptera: Braconidae: Microgastrinae) from China. Entomotaxonomia 22(3): 204–208. http://xbkcflxb.alljournal.net/xbkcflxb/ch/reader/view_abstract.aspx?file_no=20000354&flag=1

[B29] Xu WA He JH (2002a) Two new species of *Microplitis* Foerster from China (Hymenoptera, Braconidae, Microgastrinae). Acta Zootaxonomica Sinica 27(1): 153–157. 10.3969/j.issn.1000-0739.2002.01.027

[B30] Xu WA He JH (2002b) Two new species of *Microplitis* Förster (Hymenoptera: Braconidae, Microgastrinae) from China. Acta Entomologica Sinica 45(Suppl): 99–102. 10.3321/j.issn:0454-6296.2002.z1.034

[B31] Xu WA He JH (2003) Two new species of *Microplitis* Foerster from China (Hymenoptera, Braconidae, Microgastrinae). Acta Zootaxonomica Sinica 28(4): 724–728. 10.3969/j.issn.1000-0739.2003.04.032

[B32] Xu WA He JH (2006) One new species of *Microplitis* Foerster (Hymenoptera: Braconidae: Microgastrinae) from China. Entomotaxonomia 28(3): 227–230. 10.3969/j.issn.1000-7482.2006.03.009

[B33] Xu WA He JH Ye BH Zheng FQ (2000) Two new recorded species of *Microplitis* Foerster from China (Hymenoptera: Braconidae: Microgasterinae). Journal of Shandong Agricultural University (Natural Science) 31(4): 378–380. 10.3969/j.issn.1000-2324.2000.04.007

[B34] You LS Wei M (2006) Fauna Hunan HymenopteraBraconidae (I). Hunan Press of Science & Technology, Changsha, 16–28 pp.

[B35] Zhang W Song D Chen J (2017) Revision of *Microplitis* species from China with description of a new species. Zootaxa 4231(2): 296–300. 10.11646/zootaxa.4231.2.12 28187546

